# Image-Guided Thrombin Injection for Post-procedural Hematomas in Various Surgical Settings: A Case Series

**DOI:** 10.7759/cureus.94879

**Published:** 2025-10-18

**Authors:** Aws Alfahad, Dura Ibrahim, Basil Ibrahim, Rami Alfahad

**Affiliations:** 1 Radiology, American Hospital Dubai, Dubai, ARE; 2 Surgery, Manchester Foundation Trust, Manchester, GBR

**Keywords:** ct-guided thrombin injection, image-guided intervention, interventional radiology (ir), minimally invasive procedure, percutaneous thrombin injection, post-procedural hematoma

## Abstract

Post-procedural hematomas are uncommon but clinically significant complications that can lead to morbidity and mortality. Conventional management often requires surgical re-exploration or embolization, which may be technically unfeasible or high risk in patients with multiple comorbidities. Thrombin injection, although well established for pseudoaneurysm treatment, has been less frequently reported in the management of postsurgical hematomas.

We describe five patients who developed post-procedural hematomas following different interventions: robotic hernia repair, ovarian cystectomy, percutaneous nephrostomy, endoscopy, and percutaneous endoscopic gastrostomy tube insertion. Each patient was treated with image-guided thrombin injection under computed tomography or ultrasound guidance. Hemostasis was successfully achieved in all cases without thrombin-related complications, and patients recovered without the need for surgical or major endovascular intervention.

Image-guided thrombin injection is a safe, effective, and minimally invasive technique for managing selected post-procedural hematomas. This case series highlights its potential as an alternative when conventional surgical or embolization options are not feasible. Further studies are needed to standardize protocols and evaluate long-term outcomes.

## Introduction

Post-procedural bleeding is a common complication following various surgical and interventional procedures, particularly among patients with significant comorbidities. Inguinal hernia repair, pelvic operations, and nephrostomy procedures are especially prone to such vascular injuries and subsequent hematoma formation. Traditionally, management of post-procedural hematomas required surgical exploration or other invasive interventions. However, advances in interventional radiology have introduced image-guided thrombin injection as a minimally invasive and highly effective option for achieving hemostasis in such cases. Compared with conventional surgical or endovascular methods, thrombin injection offers shorter recovery, reduced procedural morbidity, and a lower risk of complications.

Multiple studies have demonstrated the safety and high success rate of thrombin injection, particularly in treating iatrogenic pseudoaneurysms and soft-tissue hematomas [1-3]. While ultrasound (US)-guided thrombin injection is well established for femoral pseudoaneurysms, its CT- or image-guided application in other postoperative or non-vascular hematomas remains relatively limited and less well documented [4-6].

Thrombin injection promotes clot formation by converting fibrinogen to fibrin, resulting in rapid and stable hemostasis. This case series presents five patients who developed post-procedural hematomas following different surgical interventions. Thrombin injection was selected as the management approach because each patient had localized, well-defined collections accessible to image-guided injection, and all were considered poor candidates for open surgical evacuation due to comorbidities or elevated bleeding risk. Traditional surgical or conservative approaches were associated with higher morbidity, prolonged recovery, or incomplete resolution in such cases. Therefore, image-guided thrombin injection provided a minimally invasive, targeted, and safe alternative that achieved rapid hemostasis and symptom relief.

## Case presentation

Case 1

**Figure 1:**
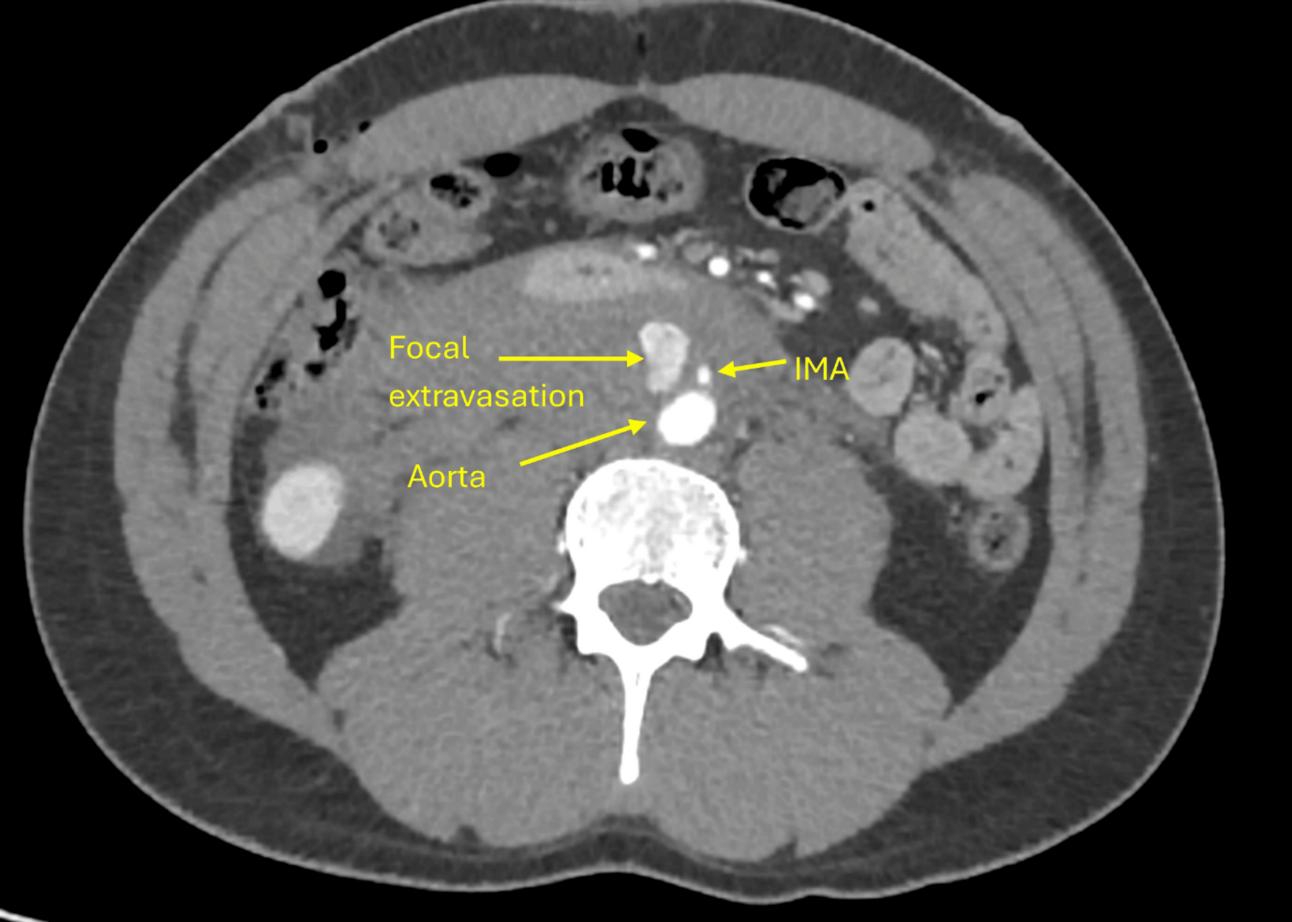
CT of the abdomen with contrast showing extravasation from the aorta with expanding hematoma at the level of the inferior mesenteric artery (IMA).

A 28-year-old male with a history of recurrent inguinal hernia underwent robotic hernia repair. Postoperatively, he developed increasing abdominal pain and mild tachycardia, raising concern for concealed hemorrhage. Given his clinical instability, a contrast-enhanced computed tomography (CT) of the abdomen was performed (Figure 1).

**Figure 2:**
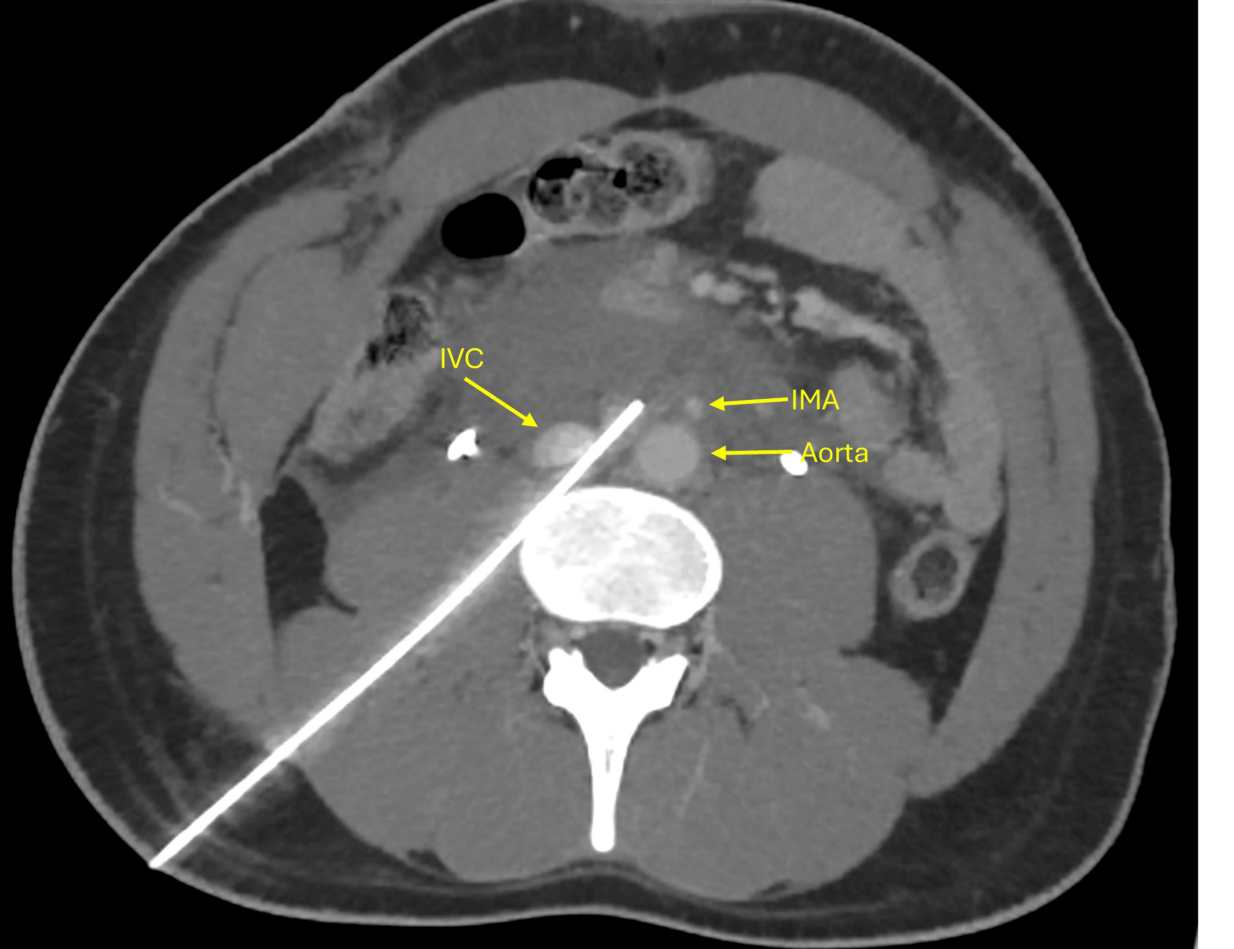
CT of the abdomen showing resolution of the hematoma with patent inferior vena cava (IVC) and inferior mesenteric artery (IMA).

The patient developed new-onset abdominal and back pain after robotic hernia repair. A CT scan performed for evaluation revealed a retroperitoneal hematoma with active extravasation from the abdominal aorta, likely due to Veress needle injury during trocar insertion. Although the patient was young, surgical re-exploration was considered high risk because the source of bleeding was a direct extravasation from the aortic wall below the level of the inferior mesenteric artery. Re-entry into the operative field after recent robotic mesh repair would have required extensive dissection through adhesions with a substantial risk of catastrophic aortic hemorrhage. Endovascular stenting was also deemed unsuitable due to the proximity of the lesion to the inferior mesenteric artery and the danger of branch occlusion. Therefore, a multidisciplinary team concluded that CT-guided thrombin injection offered the safest and most targeted means of achieving hemostasis. CT-guided thrombin injection was performed via a retroperitoneal approach and was successful in managing the bleeding (Figure 2). The patient was monitored in the intensive care unit for 48 hours following the procedure, during which he remained hemodynamically stable and had no signs of rebleeding. He was subsequently discharged in good condition and continued to recover without complications.

Case 2

**Figure 3:**
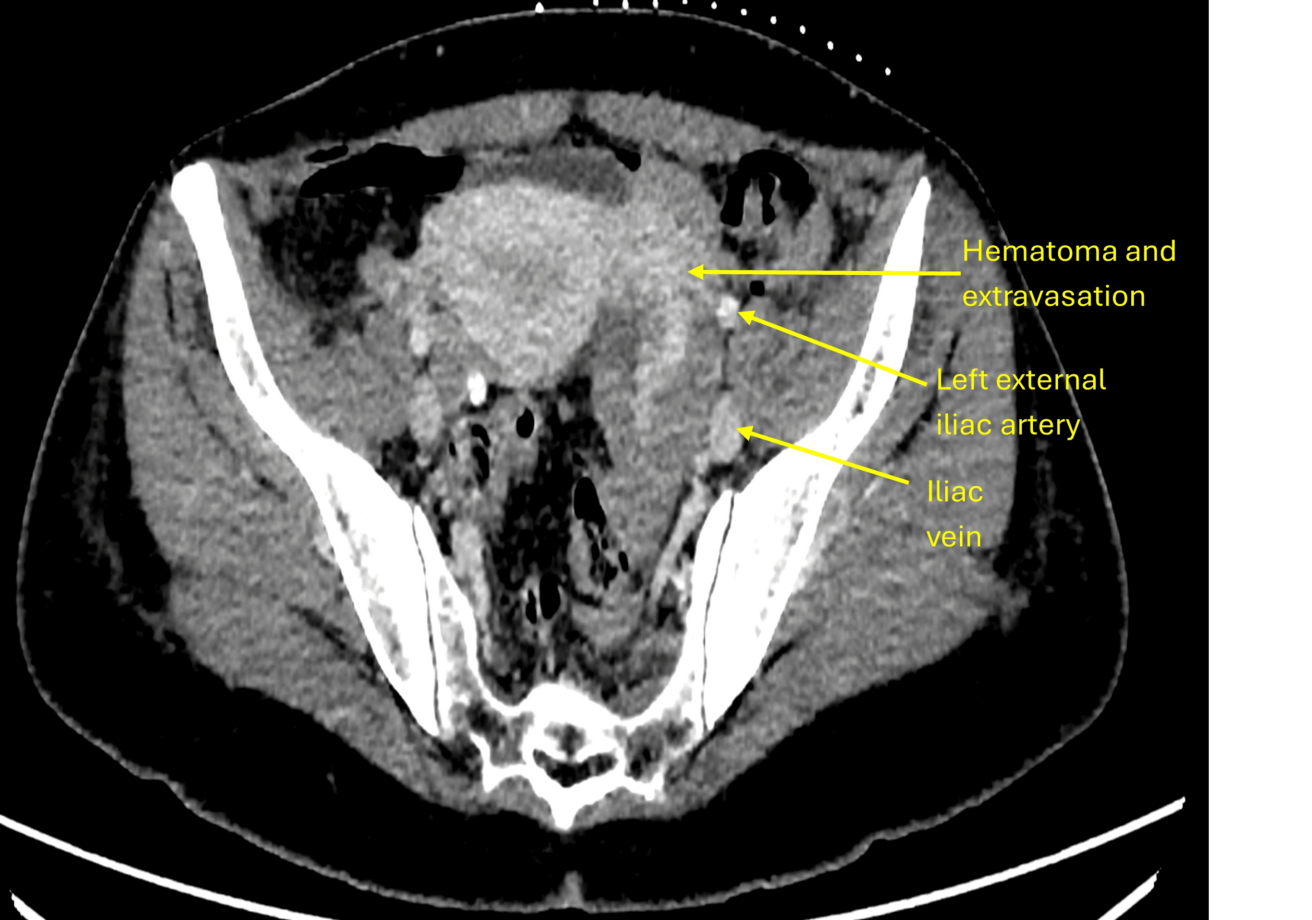
Porto-venous CT scan showing hematoma and active extravasation over the left ovarian area.

A 45-year-old female underwent robotic-assisted ovarian cystectomy with extensive adhesiolysis. On postoperative day one, she developed new abdominal pain, tenderness, and 370 mL of dark blood in the pelvic drain, raising concern for ongoing bleeding. Contrast-enhanced CT of the abdomen demonstrated a large left pelvic sidewall hematoma measuring 43 × 82 × 63 mm, with an arterial blush and progressive venous pooling (Figure 3).

**Figure 4:**
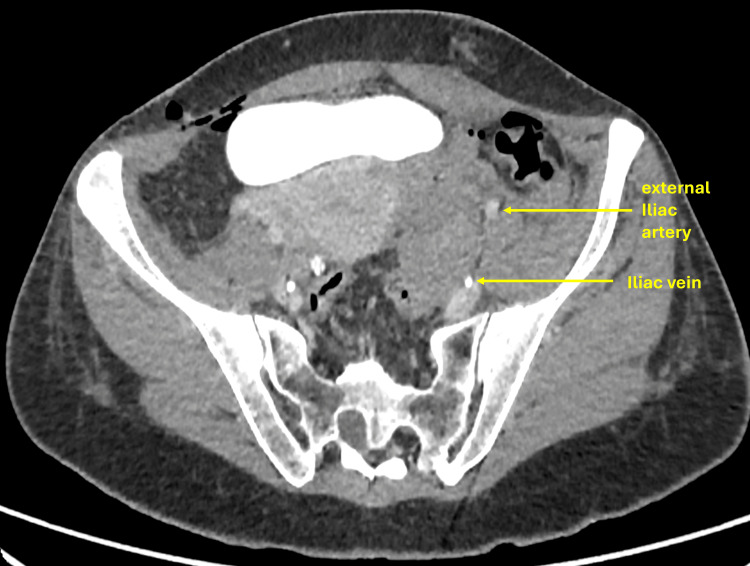
CT of the abdomen showing complete resolution of the hematoma and patent iliac arteries and veins.

Although the patient was stable, surgical re-exploration was considered high risk because the hematoma was located deep within the pelvis, adjacent to major vessels and the urinary bladder, in a recently operated field. Re-entry into the surgical site would have carried a substantial risk of hemorrhage, infection, and tissue disruption. Endovascular embolization was also deemed unsuitable due to anatomical challenges: targeting the ovarian branch of the uterine artery risked compromising uterine perfusion, while accessing the left ovarian artery, arising directly from the aorta, was technically difficult. Consequently, a minimally invasive image-guided thrombin injection was selected as the safest and most targeted alternative for achieving hemostasis. Follow-up CT confirmed resolution of bleeding with a reduction in hematoma size (Figure 4). The patient recovered uneventfully. Post-procedure CT angiography performed immediately after thrombin injection demonstrated cessation of active extravasation, confirming technical success. A follow-up CT scan obtained 48 hours later showed interval reduction in the hematoma size, consistent with effective thrombin-induced hemostasis rather than spontaneous resolution.

Case 3

**Figure 5:**
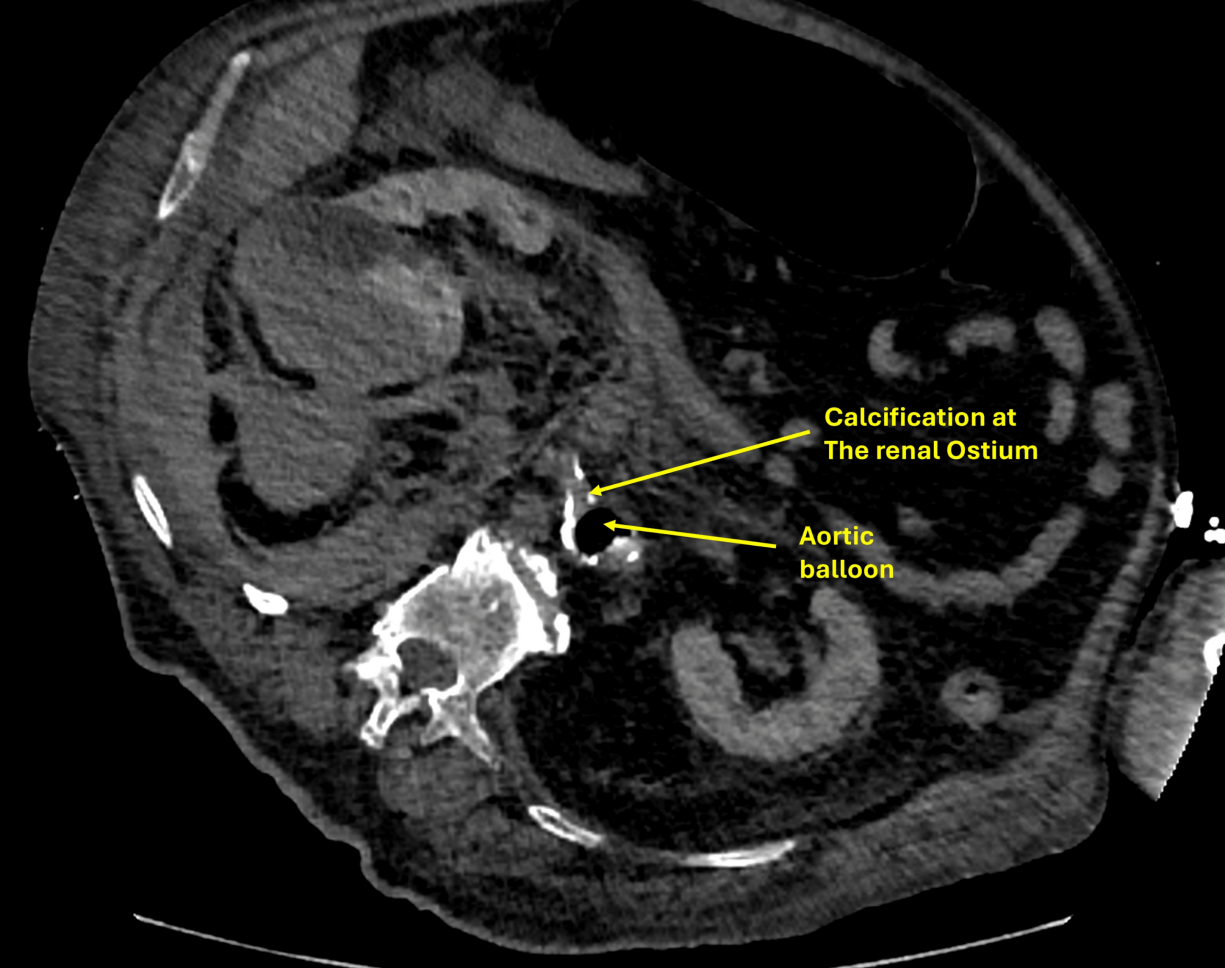
CT of the abdomen showing a calcified renal artery ostium and an intra-aortic balloon pump in place.

A 74-year-old patient presented with respiratory distress, septic and cardiogenic shock, and non-ST-segment elevation myocardial infarction (NSTEMI), with echocardiography showing left ventricular ejection fraction (LVEF) of 35% and severe mitral regurgitation. He also had acute kidney injury and urinary retention. Due to bladder outlet obstruction from stricture and prostate enlargement, retrograde access was not possible, and a percutaneous nephrostomy was planned (Figure 5).

**Figure 6:**
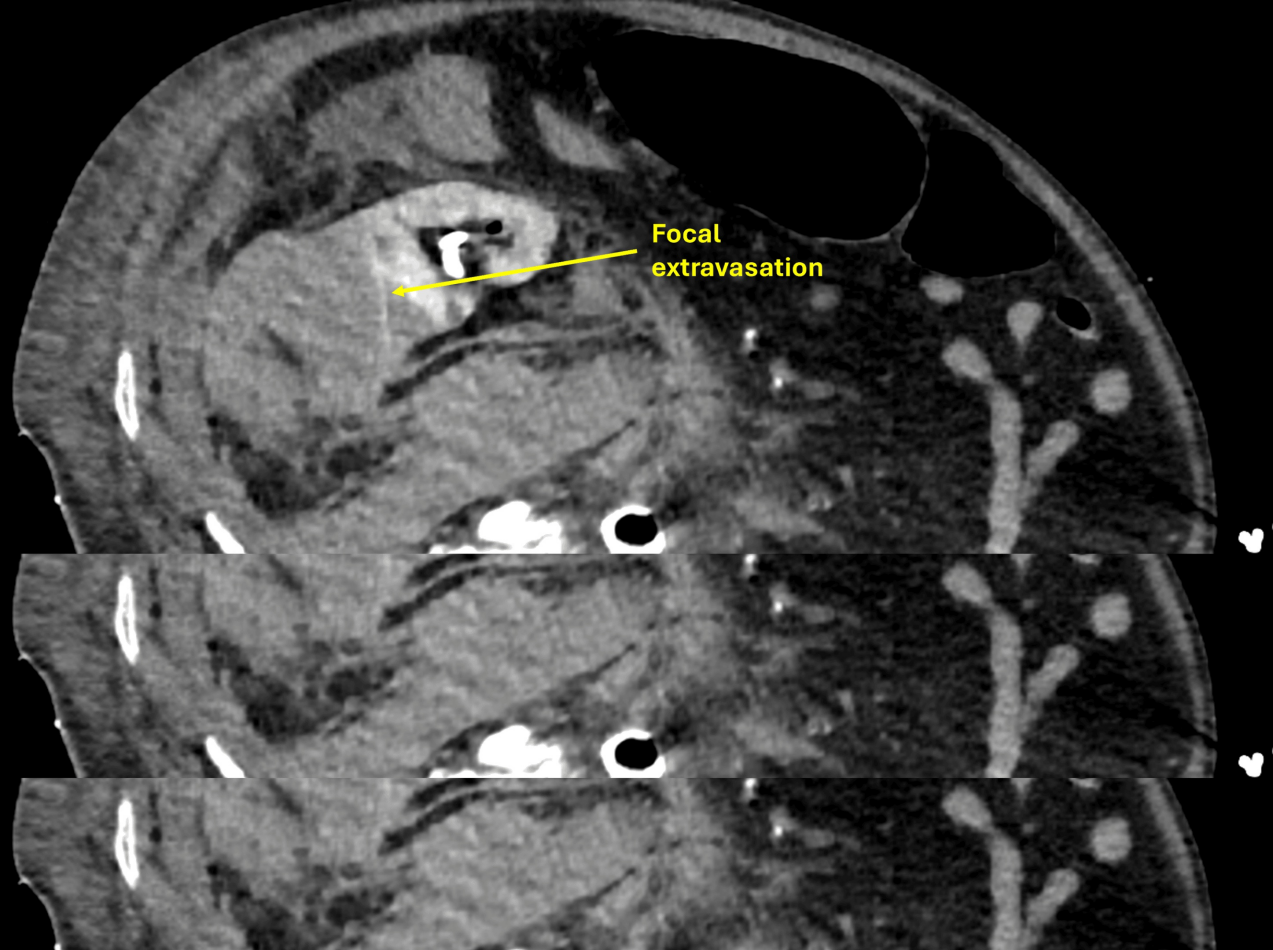
CT image showing right perinephric extravasation at the nephrostomy site.

The patient developed flank pain and a drop in hemoglobin following percutaneous nephrostomy. Post-procedure imaging revealed a large subcapsular and perinephric hematoma (8.1 × 6 cm) with active hemorrhage arising from the lower pole region of the right kidney (Figure 6). Catheter angiography was unsuitable due to the presence of an intra-aortic balloon pump and severe renal artery calcification.

**Figure 7:**
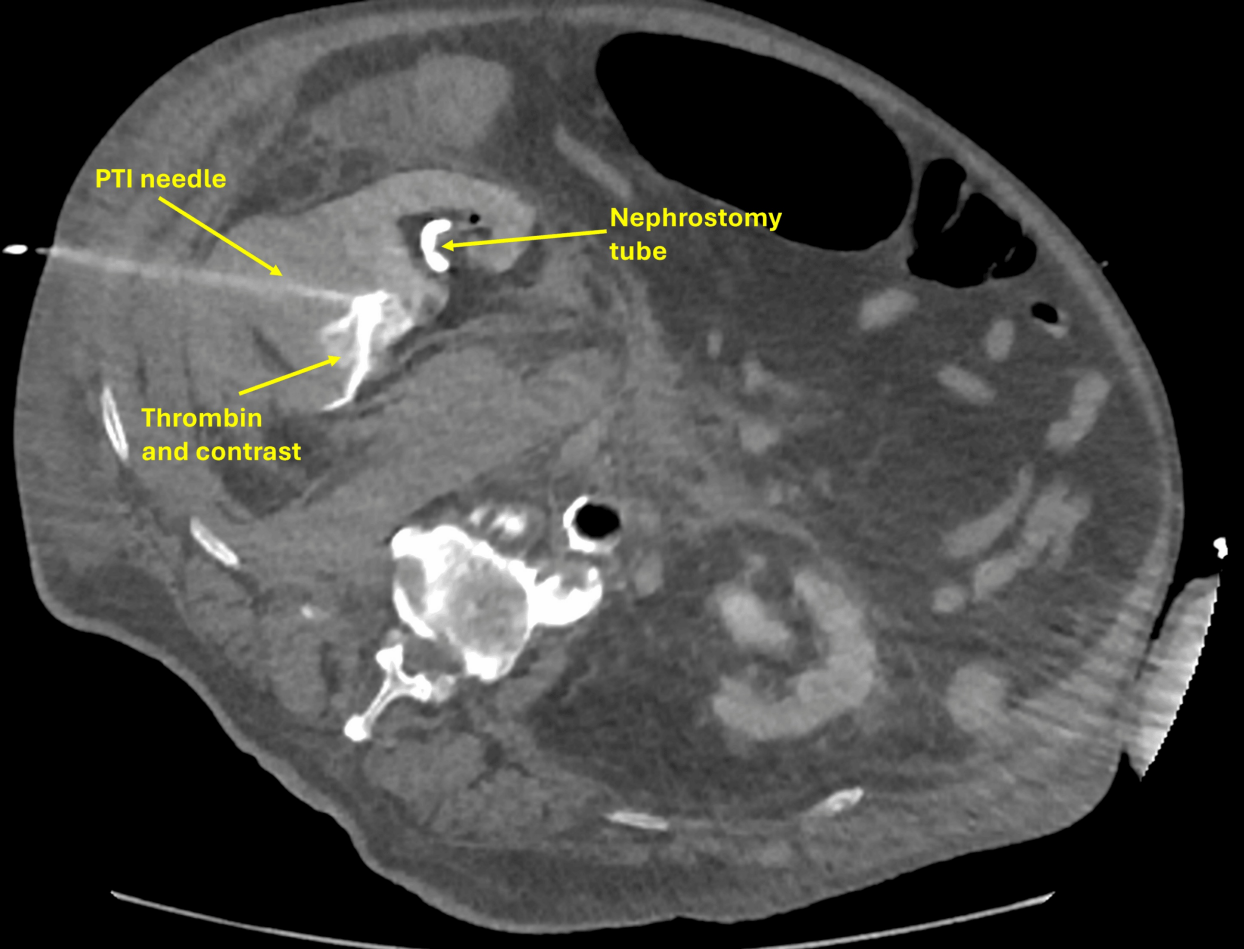
CT-guided thrombin injection into the right perinephric hematoma at the nephrostomy site. PTI: percutaneous thrombin injection.

CT-guided thrombin injection using a retroperitoneal approach was performed under aseptic conditions, injecting 10 mL of thrombin into the bleeding site, successfully controlling the hemorrhage (Figure 7). The patient recovered with improved renal function and was discharged in stable condition.

Case 4

**Figure 8:**
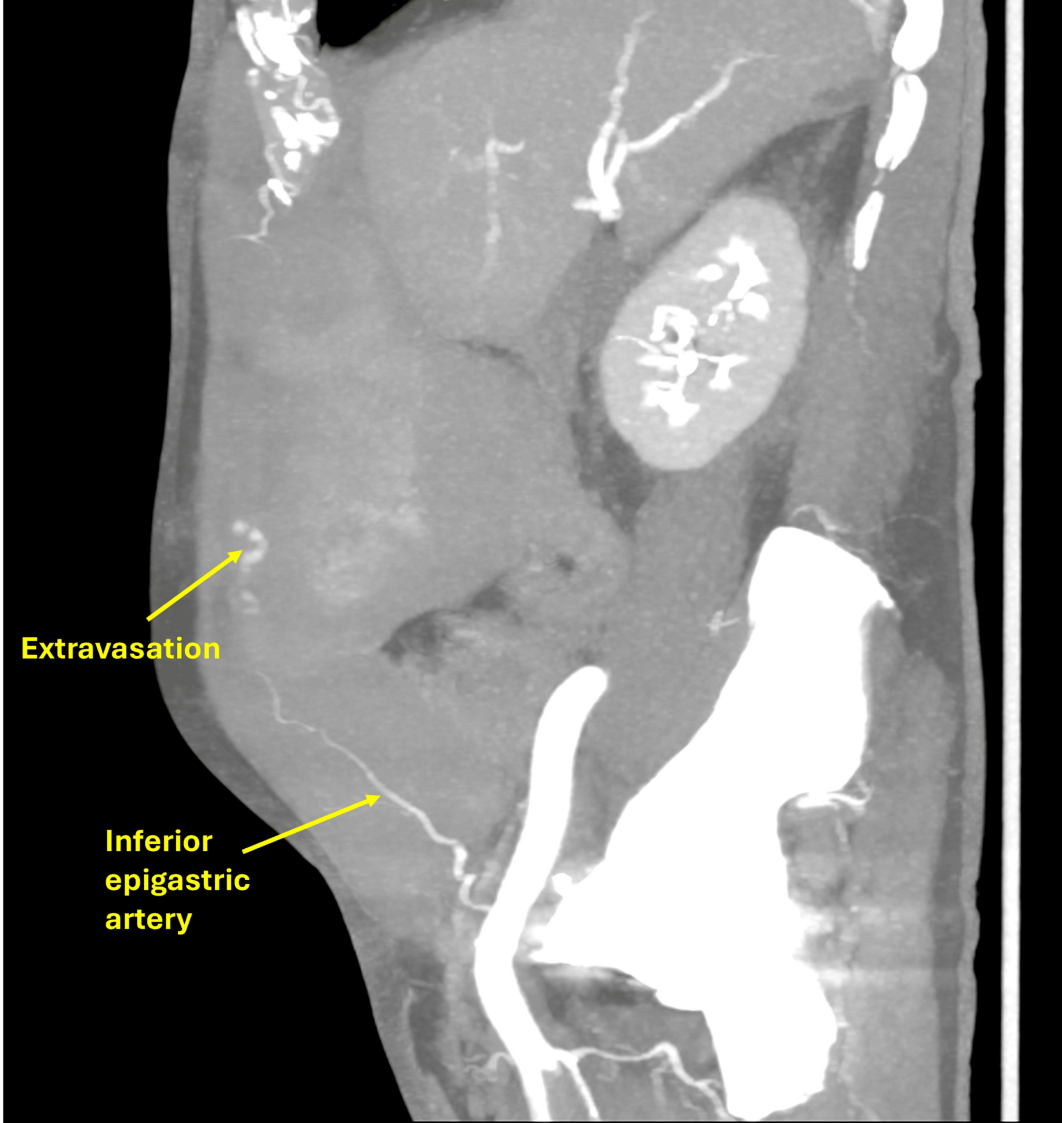
CT of the abdomen showing extravasation in the region of the right inferior epigastric artery.

A 77-year-old male on anticoagulation with subcutaneous enoxaparin 40 mg daily for atrial fibrillation developed acute abdominal pain and distension shortly after diagnostic endoscopy. Examination revealed a firm abdominal wall mass, and CT imaging demonstrated a large right rectus sheath hematoma with active extravasation from the inferior epigastric artery (Figure 8).

**Figure 9:**
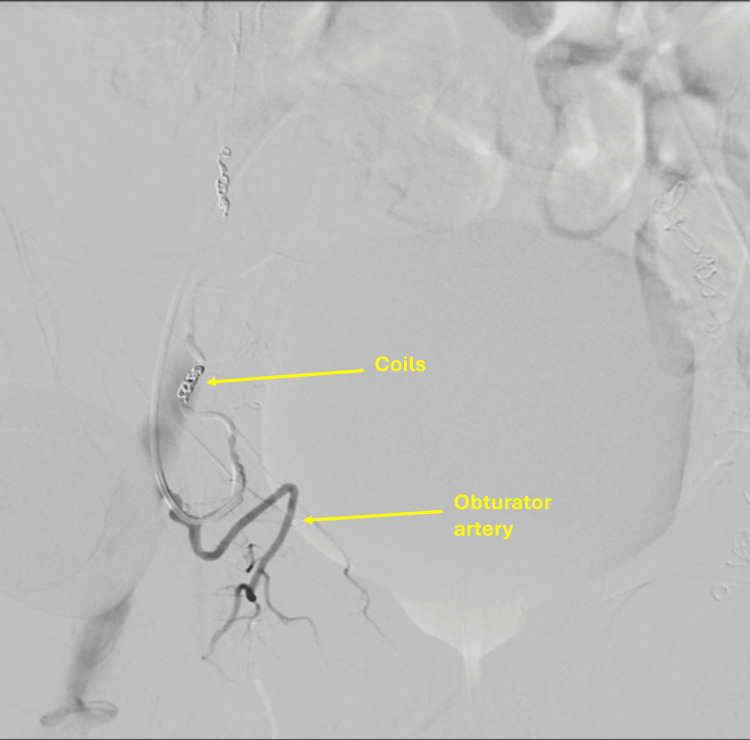
Selective embolization of the right inferior epigastric artery using 2 mm and 3 mm coils.

Although active extravasation from the inferior epigastric artery was evident on initial imaging, thrombin injection was selected as the first-line intervention due to both anatomical and clinical considerations. The bleeding vessel was small, superficial, and tortuous, making selective catheterization technically challenging and increasing the risk of non-target embolization. In addition, the patient’s advanced age and ongoing anticoagulation with enoxaparin for atrial fibrillation increased the risk of endovascular access complications. Initial thrombin injection (7,500 IU) under combined CT and ultrasound guidance significantly reduced bleeding, but mild residual vascularity persisted. Therefore, adjunct selective coil embolization was performed, and final angiography confirmed complete hemostasis with preservation of collateral flow (Figure 9). The patient remained hemodynamically stable with no procedure-related complications.

Case 5

An 86-year-old female with advanced heart failure, severe aortic stenosis, chronic kidney disease, and recurrent aspiration was admitted to the intensive care unit (ICU) for circulatory shock secondary to gastrointestinal (GI) bleeding. After stabilization, a percutaneous endoscopic gastrostomy (PEG) tube was inserted.

**Figure 10:**
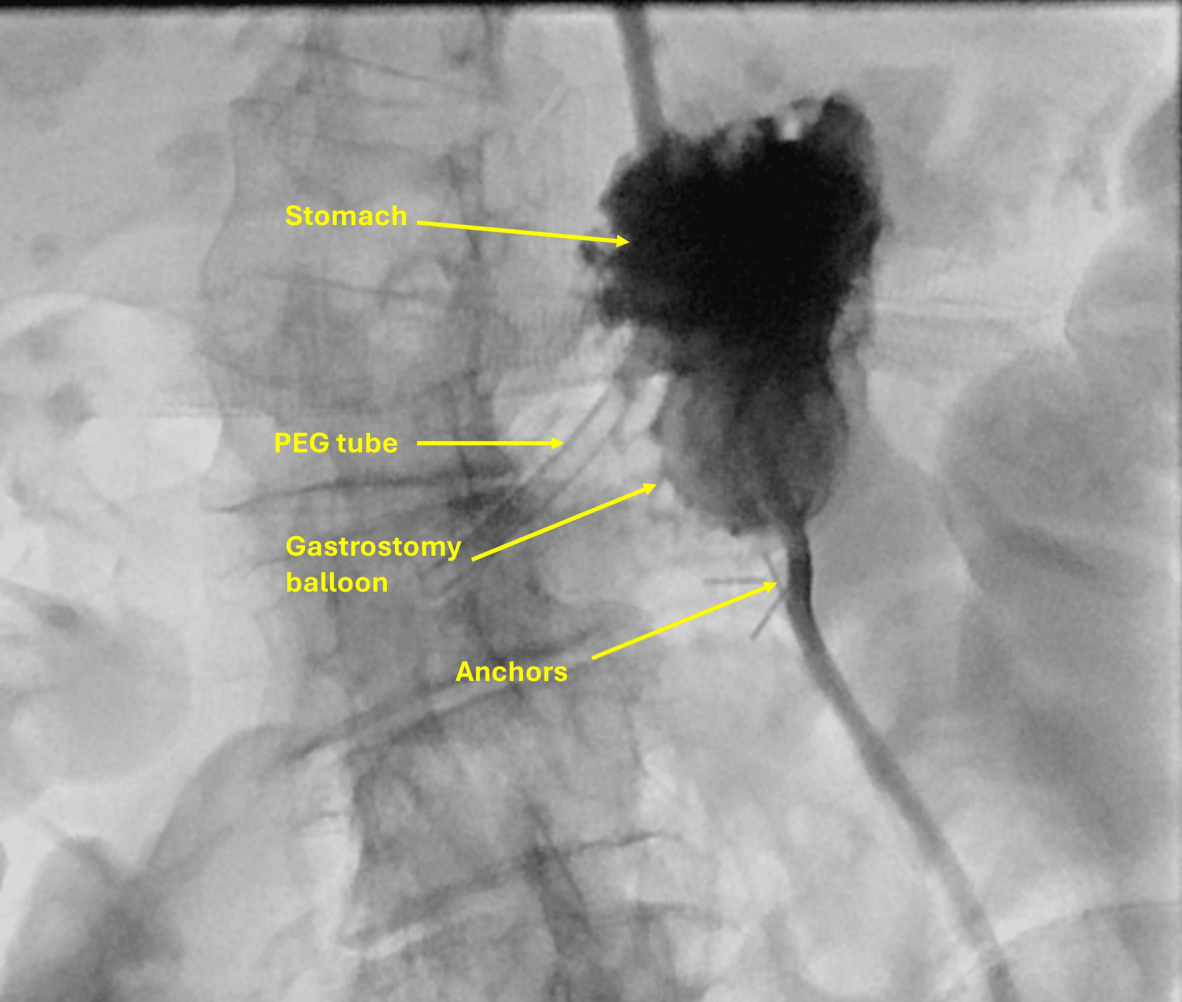
Fluoroscopy showing active bleeding from the gastrostomy tract. PEG: percutaneous endoscopic gastrostomy.

Shortly after transfer from the endoscopy suite, she developed active bleeding from the gastrostomy site with a hemoglobin drop requiring transfusion. Bedside US confirmed active bleeding at the access tract (Figure 10).

**Figure 11:**
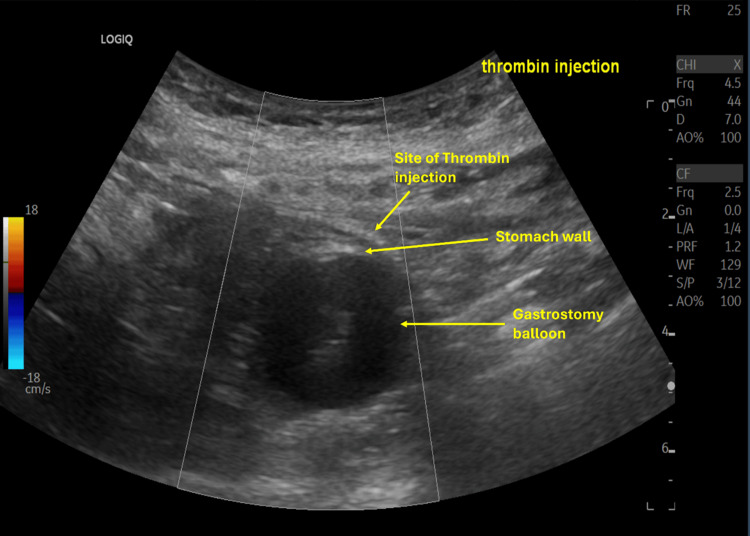
Bedside ultrasound-guided thrombin injection at the gastrostomy site demonstrating hemostasis.

Given her frailty and comorbidities, bedside US-guided thrombin injection (5,000 IU) was performed, achieving immediate hemostasis (Figure 11). The patient’s hemoglobin stabilized, and she tolerated PEG feeding without further bleeding complications.

A summary of the clinical and procedural details for all five cases, including imaging modality, thrombin dose, and outcomes, is presented in Table 1.

**Table 1:** Summary of clinical cases managed with image-guided thrombin injection. PEG: percutaneous endoscopic gastrostomy; US: ultrasound.

Case	Age/sex	Surgical procedure	Hematoma location	Imaging modality used	Thrombin dose (IU)	Additional intervention	Outcome/follow-up
1	28, M	Robotic inguinal hernia repair	Retroperitoneum (aortic extravasation)	CT-guided	10,000	None	Complete hemostasis; stable on follow-up
2	45, F	Cystectomy (gynecologic)	Pelvic/inferior epigastric artery	CT-guided	5,000	None	Hemostasis confirmed; hematoma decreased on 48-hour CT
3	74, M	Percutaneous nephrostomy	Perirenal space	CT-guided	8,000	None	Stable post-procedure; hematoma resolved
4	77, M	On enoxaparin for atrial fibrillation	Abdominal wall/inferior epigastric artery	CT + US	7,500	Adjunct coil embolization	Complete hemostasis; no complications
5	86, F	PEG insertion	Abdominal wall	US-guided	4,000	None	Stable; full recovery

## Discussion

Our series demonstrates that image-guided thrombin injection is a safe and effective alternative to embolization or surgical intervention for selected postprocedural hematomas. It is particularly useful when conventional options are technically unfeasible or contraindicated. The first reported case of CT-guided thrombin injection for retroperitoneal hematoma from aortic injury during hernia repair underscores its potential value in complex scenarios. Wider adoption and standardization of techniques and dosing protocols could further optimize safety and outcomes in interventional radiology practice.

Overall, perioperative bleeding occurs in approximately one out of every 65 non-cardiac surgical procedures, with rates being higher in individuals who have increased cardiovascular risk. Among hospitalized postsurgical patients experiencing perioperative bleeding, one-third either die during their admission or require readmission within six months [4].

Cystectomy, the surgical excision of one or both ovaries along with any related cystic structures, is commonly performed in women of reproductive age. It is typically indicated for a range of ovarian pathologies, such as benign cysts, endometriosis, and ovarian malignancies [5,6]. As with many surgical procedures, cystectomy carries a risk of postoperative complications, including bleeding.

Literature reports suggest that percutaneous nephrostomy carries an incidence of serious vascular injury of approximately 1%-2% [7-10]. However, this figure may underestimate the true rate [11]. Renal arteriovenous fistulas and pseudoaneurysms are frequently undetected at first because they are often asymptomatic and may only become apparent months or even years later [12-14]. Although uncommon, severe renal vascular injury from needle puncture can result in fatality, nephrectomy, surgical exploration, large perirenal hematomas, septicemia, transfusion diseases, and other systemic complications.

Pharmacologic agents such as aprotinin, tranexamic acid, desmopressin, and recombinant factor VIIa are utilized both prophylactically and therapeutically to control bleeding. When these medications are insufficient, alternative non-surgical techniques, including thrombin injection, may be required [15].

The application and results of percutaneous thrombin injection have been documented for various indications. Hoegger et al. reported thrombin injection following renal and hepatic biopsies, with hemostasis achieved in 94% of patients [16]. Alfahad and Alhalabi reported thrombin injection as safe and effective for PEG stoma-site bleeding [17]. Ultrasound-guided thrombin injection into visceral pseudoaneurysms has also been reported as a valuable alternative when angiographic therapy is not feasible, with low recurrence and embolization rates [18]. Additional reports describe successful treatment of hepatic artery pseudoaneurysms when embolization failed [19,20].

CT-guided thrombin injection represents a reliable and effective approach for managing post-procedural hematomas across various surgical settings. Its minimally invasive nature, combined with its ability to provide targeted hemostasis, makes it an appealing option, particularly for patients at high risk of complications. Compared with conventional surgical re-exploration, image-guided thrombin injection offers markedly reduced procedural morbidity, faster recovery, and avoidance of general anesthesia. When compared with endovascular embolization, it eliminates the risks of contrast-induced nephropathy, non-target embolization, and vascular access complications, while achieving comparable rates of hemostatic success in appropriately selected, localized hematomas. Several studies have also reported low recurrence rates and minimal adverse events, supporting its role as a safe and effective alternative to traditional methods [16-20].

## Conclusions

Our series demonstrates that image-guided thrombin injection is a safe and effective alternative to embolization or surgical intervention for selected post-procedural hematomas. It is particularly useful when conventional options are technically unfeasible or contraindicated. Although the safety and efficacy of thrombin injection have been well established in treating femoral pseudoaneurysms, its application for managing postoperative or non-pseudo-aneurysmal hematomas remains relatively uncommon and less well documented. Therefore, it is presented here as an alternative approach in complex surgical cases where conventional methods are not ideal or carry higher procedural risks. The first reported case of CT-guided thrombin injection for retroperitoneal hematoma from aortic injury during hernia repair underscores its potential value in complex scenarios. Wider adoption and standardization of techniques and dosing protocols could further optimize safety and outcomes in interventional radiology practice. Our review of the literature identified consistently high technical success rates across multiple reports, with very low recurrence and complication rates. Most published cases describe successful use of thrombin injection for femoral pseudoaneurysms, while a smaller number document its expanding role in managing visceral, parietal, and postoperative hematomas under ultrasound or CT guidance. Complications such as distal thrombosis or embolization were exceedingly rare when the technique was performed under imaging control and with appropriate dosing.

## References

[1] Liau CS, Ho FM, Chen MF, Lee YT (1997). Treatment of iatrogenic femoral artery pseudoaneurysm with percutaneous thrombin injection. J Vasc Surg.

[2] Vlachou PA, Karkos CD, Bains S, McCarthy MJ, Fishwick G, Bolia A (2011). Percutaneous ultrasound-guided thrombin injection for the treatment of iatrogenic femoral artery pseudoaneurysms. Eur J Radiol.

[3] Gupta A, Naranje P, Vora Z (2022). Intranodal lipiodol injection for the treatment of chyle leak in children - a preliminary experience. Br J Radiol.

[4] Smilowitz NR, Gupta N, Ramakrishna H, Guo Y, Berger JS, Bangalore S (2017). Perioperative major adverse cardiovascular and cerebrovascular events associated with noncardiac surgery. JAMA Cardiol.

[5] Mobeen S, Apostol R (2025). Ovarian cyst. StatPearls.

[6] (2006). Overview: Ovarian cysts. Cologne (DE): InformedHealth.org.

[7] Newhouse JH, Pfister RC (1981). Percutaneous catheterization of the kidney and perinephric space: trocar technique. Urol Radiol.

[8] Stables DP, Ginsberg NJ, Johnson ML (1978). Percutaneous nephrostomy: a series and review of the literature. AJR Am J Roentgenol.

[9] Ho PC, Talner LB, Parsons CL, Schmidt JD (1980). Percutaneous nephrostomy: experience in 107 kidneys. Urology.

[10] Günther R, Alken P, Altwein JE (1979). Percutaneous nephropyelostomy using a fine-needle puncture set. Radiology.

[11] Schilling A, Goettinger H, Marx FJ, Schueller J, Bauer HW (1981). A new technique for percutaneous nephropyelostomy. J Urol.

[12] Pfister RC, Newhouse JH (1979). Interventional percutaneous pyeloureteral techniques. II. Percutaneous nephrostomy and other procedures. Radiol Clin North Am.

[13] Hildell J, Aspelin P, Sigfússon B (1980). Percutaneous nephrostomy. Aspects on clinical application. Acta Radiol Diagn (Stockh).

[14] Saxton HM, Ogg CS, Cameron JS (1972). Needle nephrostomy. Br Med Bull.

[15] Koh MB, Hunt BJ (2003). The management of perioperative bleeding. Blood Rev.

[16] Hoegger MJ, Middleton WD (2022). Ultrasound-guided thrombin injection for the treatment of bleeding following kidney and liver biopsies. J Ultrasound Med.

[17] Alfahad A, Alhalabi R (2024). Ultrasound (US)-guided percutaneous thrombin injection for stoma-site bleeding after PEG tube insertion: a case series and review of the literature. CVIR Endovasc.

[18] Barge JU, Lopera JE (2012). Vascular complications of pancreatitis: role of interventional therapy. Korean J Radiol.

[19] Francisco LE, Asunción LC, Antonio CA, Ricardo RC, Manuel RP, Caridad MH (2010). Post-traumatic hepatic artery pseudoaneurysm treated with endovascular embolization and thrombin injection. World J Hepatol.

[20] Dambrin C, Marcheix B, Birsan T (2005). Posttraumatic pseudoaneurysm of the hepatic artery: treatment with ultrasound-guided percutaneous transhepatic thrombin injection. J Trauma.

